# LEF1-AS1 contributes to proliferation and invasion through regulating miR-544a/ FOXP1 axis in lung cancer

**DOI:** 10.1007/s10637-018-00721-z

**Published:** 2019-02-08

**Authors:** Ansheng Wang, Chengling Zhao, Yuan Gao, Guixin Duan, Yuming Yang, Bo Fan, Xiaojing Wang, Kangwu Wang

**Affiliations:** 1 Departments of Thoracic Surgery, The First Affiliated Hospital of Bengbu Medical College, No.287 Changhuai Road, Bengbu City, Anhui Province 233004 People’s Republic of China; 2 Department of Respiratory Diseases, The First Affiliated Hospital of Bengbu Medical College, No.287 Changhuai Road, Bengbu City, Anhui Province 233004 People’s Republic of China

**Keywords:** Lung cancer, Long non-coding RNAs, LEF1-AS1, LEF1-AS1/miR-544a/FOXP1 axis

## Abstract

Long non-coding RNAs (lncRNAs) are increasingly recognized as important regulators in tumor development. This study aims to investigate the potential role oflncRNALEF1-AS1, in the progression of lung cancer. Quantitative real-time PCR (qRT-PCR) and western blot assays showed that LEF1-AS1 was upregulated while miR-544a was downregulated in lung cancer specimens and cells. Overexpression of LEF1-AS1 led to the enhancement of cell proliferation and invasion, revealed by CCK-8 assay and transwell assay. A negative correlation was found between LEF1-AS1 and miR-544a. BLAST analysis and dual-luciferase assay confirmed that FOXP1 is a downstream effector of miR-544a. Therefore, the LEF1-AS1/miR-544a/FOXP1 axis is an important contributor to lung cancer progression. Collectively, our novel data uncovers a new mechanism that governs tumor progression in lung cancer and provides new targets that may be used for disease monitoring and therapeutic intervention of lung cancer.

## Introduction

Lung cancer is the most common cause of cancer-related deaths in the globe and accounts for an estimated 1.6 million deaths each year [1]. The majority (85%) of lung cancer patients suffers from non-small cell lung cancer, including adenocarcinomas and squamous cell carcinomas [2]. Due to the high mortality and morbidity of lung cancer, it is imperative to understand the underlying molecular mechanism of lung cancer tumorigenesis to develop new prognostic markers and effective therapeutic strategies [3–5].

LncRNAs, defined as oligonucleotides with lengths of greater than 200 nucleotides [6, 7], are transcribed by RNA polymerase II and frequently originate from intergenic regions. LncRNAs make up a considerable component of the mammalian transcriptome [6], which do not possess substantial open reading frames and can be spliced, capped and polyadenylated [8, 9]. Fundamentally, the location, abundance and distribution of lncRNAs throughout the genome provides the organism with an additional method to control the expression of thousands of proteins, by transcriptional and posttranscriptional modifications. Recently, Long non-coding RNAs (lncRNAs) have recently been uncovered in the human genome and found to play a pivotal role in regulating many oncogenic pathways in various cancer types, including those found in lung cancers 6. Many lncRNAs have been shown to play crucial roles in at least one hallmark of cancer and can behave as either oncogenes or tumor suppressors [10, 11].

Human lymphoid enhancer-binding factor 1 antisense RNA 1 (LEF1-AS1) is a newly discovered lncRNA located on the plus strand of chromosome 4 [12]. LEF1-AS1 was previously shown to be upregulated in glioblastoma (GBM) tissues and its dysregulation was postulated to correlate with poor overall survival in patients [13]. Additionally, knockdown of LEF1-AS1 demonstrated tumor-suppressing effects, such as lowering cancer cell proliferation, invasion and migration. These findings uncovered a role of LEF1-AS1 as a target oncogene in GBM, but failed to confirm the underlying signaling mechanism. Here, we show that LEF1-AS1 promotes proliferation and invasion in lung cancer by regulating the miR-544a/ FOXP1 axis. These findings may provide a valuable support for LEF1-AS1 used as a potential target for the therapy of lung cancer, as well as establish a foundation for LEF1-AS1 could serves as a novel target for anti-cancer drug in future.

## Methods

### Clinical tissue specimens

A total of 48 pairs of lung cancer tissues and adjacent normal tissue were acquired from The First Affiliated Hospital of Bengbu Medical College between Jan 2012 and Sep 2014. The study protocol was approved by the Ethics Committees of The First Affiliated Hospital of Bengbu Medical College. All patients provided written informed consent. Samples were stored at −80 °C until use.

### Cell lines and culture

The normal human lung epithelial cell, BEAS-2B, and human lung cancer cell lines, including H1299, A549, H1975 and SPC-A-1, were purchased from ATCC (Manassas, VA). Cells were cultured in RPMI 1640 medium supplemented with 10% fetal bovine serum in humidified condition with 95% air and 5% CO_2_ at 37 °C.

### Oligonucleotides transfection

siRNA against LEF1-AS1 (Si-LEF1-AS1), short-hairpin RNA plasmid specific to LEF1-AS1 (sh- LEF1-AS1), miR-544a inhibitor, miR-544a mimics, and their controls were synthesized by GenePharma (Shanghai, China). Oligonucleotide transfection were performed using Lipofectamine 2000 (Invitrogen, Carlsbad, CA) according to the manufacturer’s protocol. The sequence of siRNA for LEF1-AS1 and Control: Si-LEF1-AS1, sense 5′-GGCCAAGGAAUUUACUUAUUU-3′, antisense 3′-UUCCGGUUCCUUAAAUGAAUA-5′; Control: sense: 5′ - GGCCGAGGCTCAATGUTTUUU -3′, antisense: 5′ - UUTTGGUUGGCUAAAGCATUA -3′;

### BLAST alignment

NCBI’s BLAST suite was used for alignment searches. The top search results with an value <0.01 was reported. RNA transcripts were allowed to have multiple exons aligning to different non-contiguous regions of a chromosome. We proceeded our study using miR-544a, a miRNA with a high affinity to LEF1-AS1.

### qRT-PCR

Total RNA were isolated from tissues and cells using the miRNeasy Mini Kit (Qiagen, Valencia, CA, USA) according to the manufacturer’s instructions. Quality and concentration of RNA were evaluated with NanoDrop 2000 (Thermo Fisher, Wilmington, DE, USA). cDNA was synthesized by TransScript first-strand cDNA synthesis SuperMix (TransGen, Beijing, China). RT-PCR assay was carried out by ABI prism 7500 sequence detection system (Applied Biosystems Life Technologies) using SYBR green qPCR SuperMix (Applied Biosystems Life Technologies, Foster, CA, USA). The expression of genea was quantified using the 2^−ΔΔCt^ (cycle threshold), method and the expression levels of miRNA and lncRNA/target gene were normalized by U6 and GADPH, respectively. The primer sequenceswere showed as follows:: LEF1-AS1, forward: 5’-GGGCCCCTTTGTGTGACTAA-3′; reverse, 5’-ACCTGCGCTAAGAACTGAGG-3′; miR-544a, forward: 5′- TAAAAGCTGGCAACTGTCTAA-3′, reverse, 5′- ATTAGTAGGAAATTGCTGCAG-3′; GAPDH, forward, 5′-TCGACAGTCA GCCGCATCTTCTTT-3′, reverse, 5′-ACCAAATCCGTTGACTCCGACCTT-3′.

### Luciferase reporter assay

LEF1-AS1 cDNA fragment that encompassed microRNA binding sites was inserted into the pmirGLO plasmids (Promega, Madison, WI, USA). Mutant LEF1-AS1 (pmirGLO- LEF1-AS1-MUT) generated by site-directed mutagenesis PCR with platinum pfx DNA polymerase was which served as the negative control. Target miR-544a mimics or miR-NC mimics and luciferase reporter plasmids and were cotransfected into cells using Lipofectamine 2000. At 48 hafter transfection, relative luciferase activity was measured in a luminometer by Dual-Luciferase Reporter Assay System (Promega).

### Cell proliferation assay

Cell Counting Kit-8 (CCK- 8; Dojindo, JPN) was used to assess cell proliferation. A549 and H1299 cells transfected with si-NC, si-LEF1-AS1, miR-544a inhibitor or si- LEF1-AS1+ miR-544a inhibitor were collected and seeded into 96-well plates. After 24, 48 72 or 96 h, 10 μl of CCK-8 assay reagent was added to each well. After incubation for 2 h, DMSO was added and the absorbance was measured using an enzyme immunoassay analyser (Bio-rad, Hercules, CA, USA).

### Cell migration and invasion assay

Wound healing assay and transwell assay were performed to measure the migration and invasion ability of breast cancer cells respectively. For wound healing assay, when A549 CSC and H1299 CSC cells were cultured to 90% confluence in 96-well plates, the medium was removed and a gap was made by enforcing the sterile pipette tip on the monolayer cells. The width of the wound gap at 24 h was acquired and normalized to initial distance at 0 h. Migration rate was calculated using the following formula: migration rate = migration distance/ original distance. For transwell assay, A549 CSC and H1299 CSC cells were suspended in 200 ml serum-free DMEM and seeded in chambers (8 mm, BD Biosciences) coated with BD BioCoat Matrigel. After incubation, the non-invaded cells on the upper membrane surface were removed with a cotton tip. The cells on membrane were fixed and stained by violet crystalline.

### Western blot analysis

The total protein was extracted using the RIPA buffer (Sigma–Aldrich, St. Louis, MO) supplemented with protease inhibitors cocktail (Roche, Diagnostics, Mannheim, Germany). Protein concentration was measured using BCA assay. Proteins were separated by SDS–PAGE, followed by being transferred to PVDF membrane (Millipore, Bedford, MA). After blocked with 5% non-fat milk, the membrane was incubated with the primary antibodies, including anti-FOXP1 (1:1000, Abcam, Cambridge, MA), anti-GADPH (1:1000, Abcam) d. After washing with TBST, PVDF membrane was incubated with HRP-conjugated goat anti-rabbit IgG (Abcam) at room temperature for 2 h. Finally, the films were developed using ECL detection kit (Beyotime Biotechnology, Shanghai, China).

### Lentivirus construction and infection

Construction of a lentiviral vector expressing LEF1-AS1-shRNA was performed by Shanghai Genechem. LEF1-AS1-shRNA was inserted into pFU-GW-RNAi vector carrying the green fluorescent protein (GFP) reporter driven by the U6 promoter. A549 cells were seeded into 6-well plates with 2 × 10 [5] cells per well. After 12 h, A549 cells were infected with Lv-shRNA-NC or Lv-shRNA- LEF1-AS1 at 10 MOI, respectively. Culture medium was changed at 12 h after infection.

### Animal experiments

All animal experiments were performed according to protocols and approved by the Institutional Animal Care and Use Committee of The First Affiliated Hospital of Bengbu Medical College. Briefly, 1 × 10 [6] A549 cells infected with lentivirus carrying sh- LEF1-AS1 or sh-NC were subcutaneously injected. Tumor size was measured by a caliper every 3 days. Tumor volume was calculated using the following formula: volume = 0.5 × length × width [2].

### Immunohistochemical staining

Tumor tissue were sectioned at the thickness of 5 μm and embedded in paraffin. To perform immunohistochemical staining, tissues were dewaxed and rehydrated in graded concentrations of xylene/alcohol. Antigen retrieval was performed in citrate buffer (pH 6.0) and heating at 121 °C. Sections were then blocked in goat serum (Boster, Wuhan, China) for 30 min at room temperature. Ki67 antibody (Bioss Antibodies, Inc., 1:200) was used to incubate the sections overnight at 4 °C. For TUNEL assay, Colorimetric TUNEL Apoptosis Assay Kit (Beyotime, Shanghai, China) was used to incubate the sections at 37 °C for 60 min. Following, Polink-1 HRP DAB Detection System One-step polymer detection system (ZSGB-BIO, Beijing, China) were added to the section and incubated for 20 min at room temperature. Hematoxylin was lastly used to stain the nucleus.

### Statistical analysis

All the statistical data are presented as the means ± S.D. Two-tailed Student’s t test or one-way ANOVA followed by the LSD post hoc test was performed for comparisons between groups. Expression correlation assays were analyzed using Pearson’s coefficient correlation. Differences in patient survival were performed using the Kaplan-Meier method and analyzed by log-rank test. A value of *P* < 0.05 was considered to be statistically significant.

## Results

### LEF1-AS1 upregulation in lung cancer is associated with the poor survival of patients

**Fig. 1:**
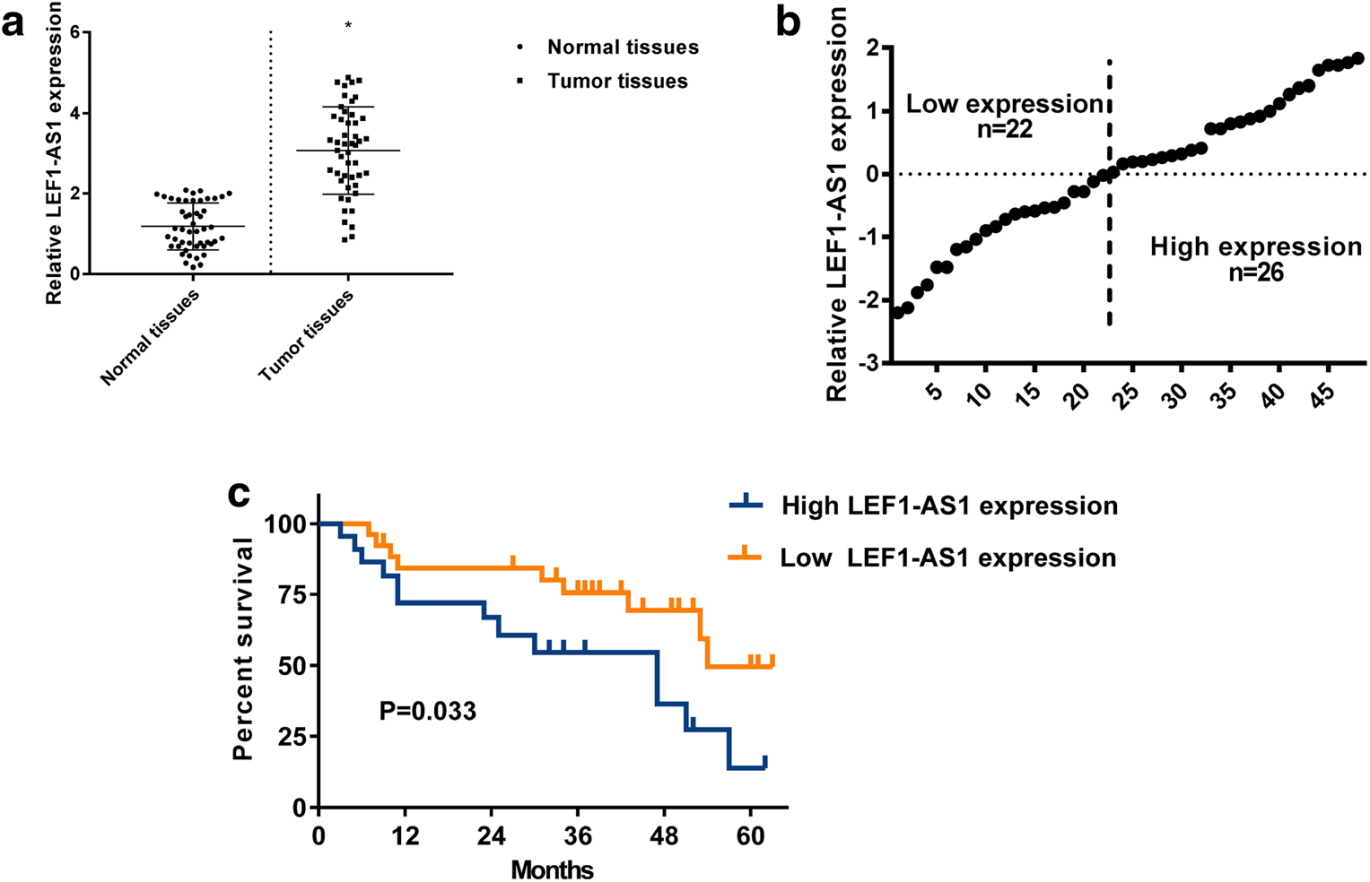
**LEF1-AS1 upregulation is associated with lung cancer aggressiveness**. **a**, qRT-PCR analysis of LEF1-AS1 expression, suggesting a higher LEF1-AS1 expression in tumor tissue, compared to normal tissue from lung cancer patients (stage I and II) (*N* = 48). * *p* < 0.05. **b**, Grouping of patients according to LEF1-AS1 expression. High-expression patients or low-expression patients were grouped based on average LEF1-AS1 expression. **c**, Overall survival curve of patients with high or low LEF1-AS1 expression. *p < 0.05

To explore the role of LEF1-AS1 in lung cancer, qRT-PCR analysis was first performed to detect the expression of LEF1-AS1 in lung cancer specimens and adjacent normal tissue from patients (*N* = 48). We found thats, LEF1-AS1 expression was significantly higher in tumor tissues comparing with the adjacent tissues (*P* < 0.05, Fig. 1a). Next, we divided the patients into two groups based on the LEF1-AS1 expression, using the average LEF1-AS1 level as the threshold (Fig. 1b). Survival analysis of showed that the overall survival of patients with high LEF1-AS1 expression was much poorer than those with low LEF1-AS1 expression (Fig. 1c), suggesting that high LEF1-AS1 was associated with lung cancermalignancy and poor survival of patients.

### LEF1-AS1 promotes lung cancer proliferation and invasion

**Fig. 2:**
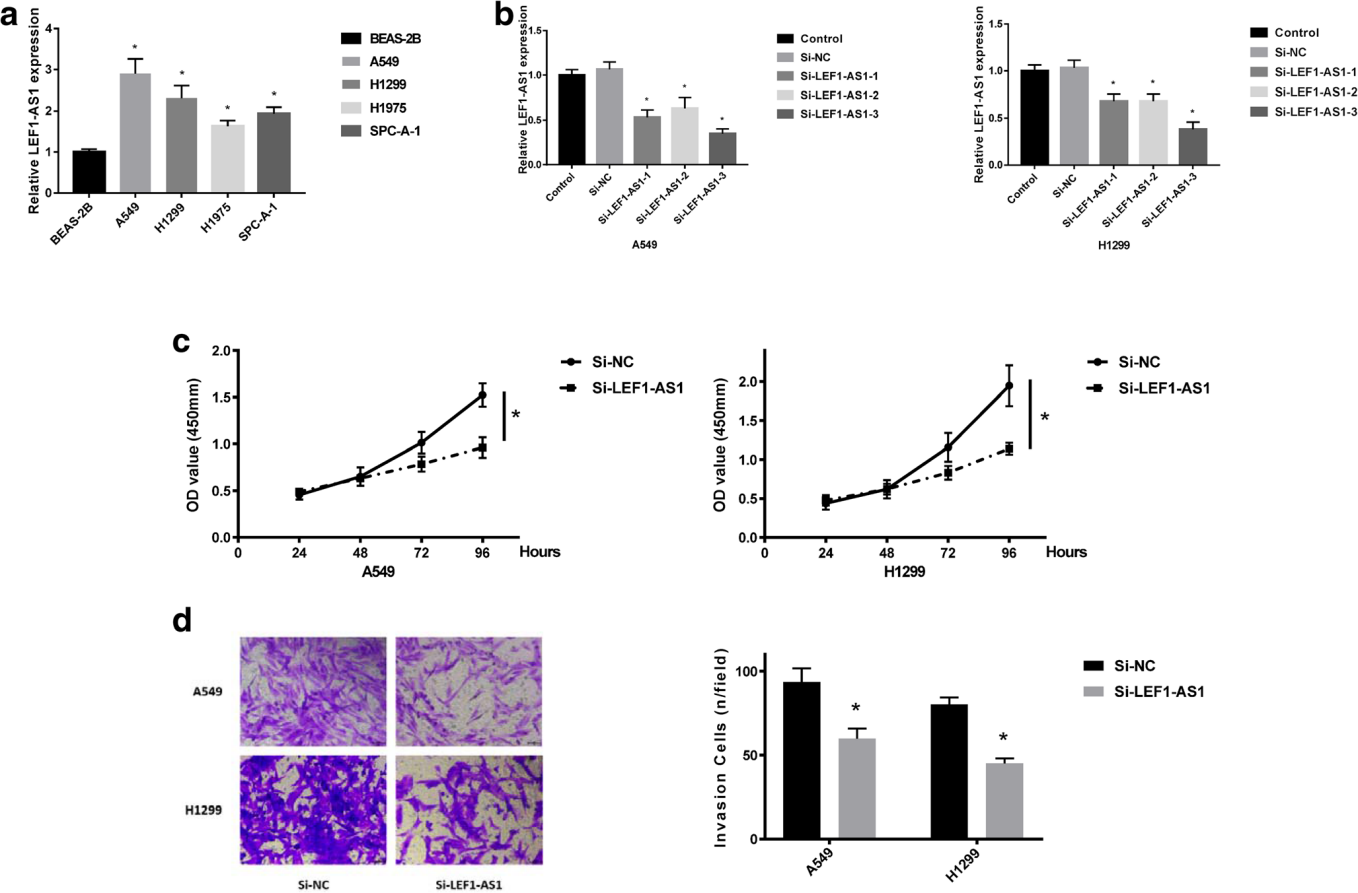
**LEF1-AS1 promotes lung cancer proliferation and invasion**. **a**, Comparison of LEF1-AS1 expression in lung cnacer cells using qRT-PCR analysis. **b**, Change of LEF1-AS1 levels after A549 cells or H1299 cells were transfected with three si-LEF1-AS1s (si-LEF1-AS1–1, si-LEF1-AS1–2 and si-LEF1-AS1–3). **c**. Proliferation assay of A549 cells or H1299 cells after si-LEF1-AS-31 transfection. Cells transfected with si-NC were used as a control. **d**, Representative staining images and quantitative analysis of invaded cells in the lower chamber of transwell assay, comparing the invasive ability of A549 or H1299 cells transfected with si-NC or si-LEF1-AS1. * *P* < 0.05

To further confirm the role of LEF1-AS1 in lung cancer, we analyzed the expression of LEF1-AS1in lung cancer A549, H1299, H1975 and SPC-A1 cells, with BEAS-2B cells as the control. Consistent with the upregulation of LEF1-AS1 in lung cancer tissues, LEF1-AS1 expression was also significantly increased in the four tumor cells, compared to BEAS-2B cells (Fig. 2a). Especially, A549 and H1299 cells demonstrated the most prominent LEF1-AS1 upregulation, thus which were selected for subsequent studies. LEF1-AS1 silencing was achieved by transfecting three si-LEF1-AS1s into A549 and H1299 cells. Obviously, all three siRNAs induced pronounced LEF1-AS1 downregulation. Since si-LEF1-AS1–3 showed the most marked LEF1-AS1 downregulation, si-LEF1-AS1–3 was used as the lead siRNA to suppress LEF1-AS1 expression in lung cancer cells (Fig. 2b**)**. In A549 and H1299 cells, si-LEF1-AS1 transfection significantly attenuated cell proliferation (Fig. 2c**)** and invasion (Fig. 2d**)**. These data confirmed the tumor-promoting role of LEF1-AS1 in lung cancer.

### miR-544a is the target of LEF1-AS1

**Fig. 3:**
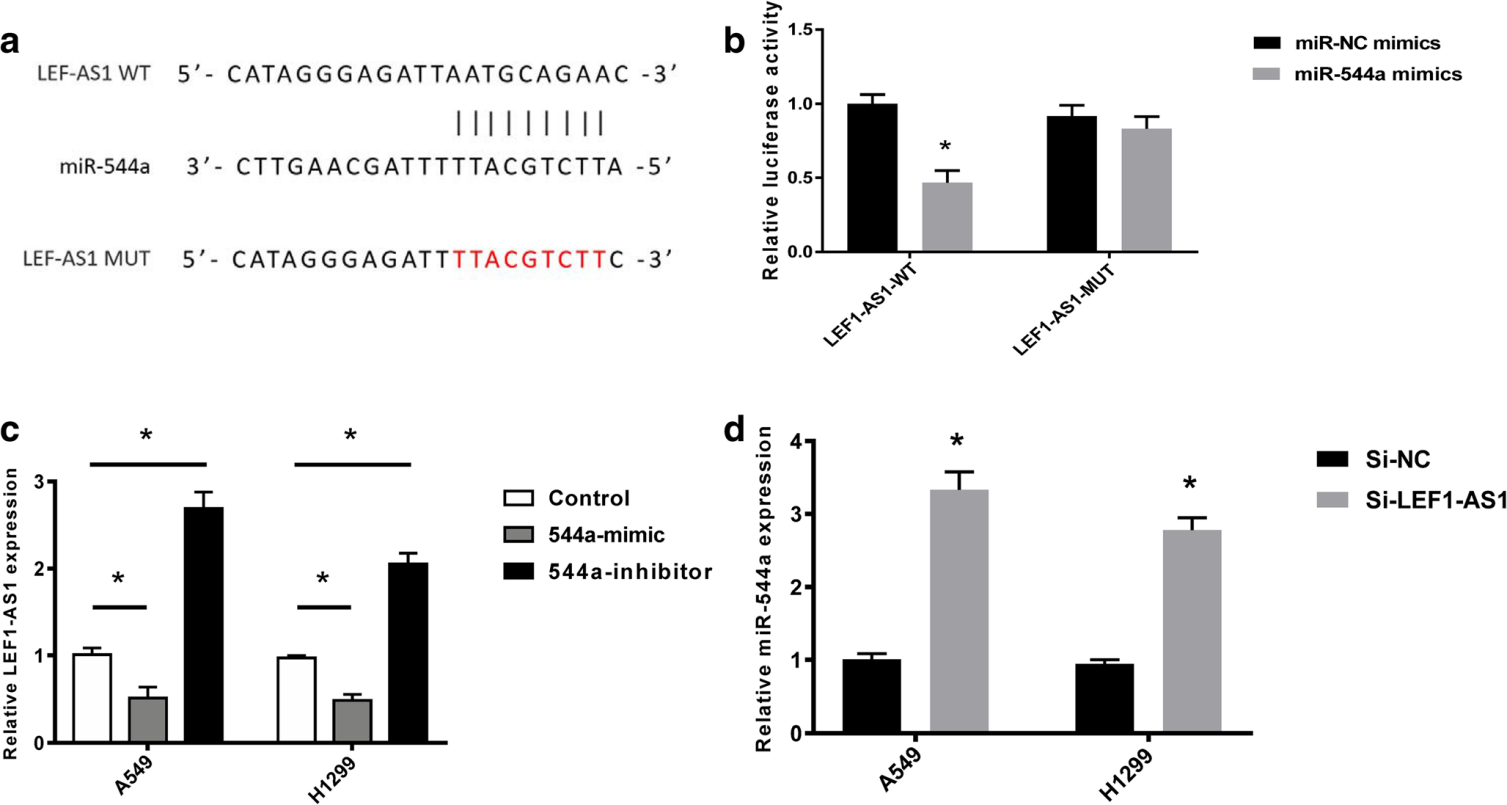
**miR-544a is the target of LEF1-AS1. a**, BLAST analysis, which identified a binding site between wild-type LEF1-AS1 (LEF1-AS1-WT) and miR-544a. LEF1-AS1 mutant (LEF1-AS1-MUT) sequence was designed for further luciferase study. **b**, Cells were transfected indicated vectors and dual-luciferase assay was used to study the relationship between miR-NC/miR-544a and LEF1-AS1-WT or LEF1-AS1-MUT. **c**, Analysis of LEF1-AS1 expression using qRT-PCR in A549 and H1299 after 544a mimic or 544a-inhibitor transfection. **d**, analysis of 544a expression in A549 or H1299 cells after si-LEF1-AS1 or si-NC infection. * p < 0.05

To clarify the mechanism of LEF1-AS1 in lung cancer regulation, we performed BLAST analysis and identified a binding site between LEF1-AS1 and miR-544a (Fig. 3a). Further, a mutated LEF1-AS1 sequence was designed to explore the specificity of the interaction between LEF1-AS1-WT and miR-544a (Fig. 3a). As shown in Fig. 3b, the result of dual-luciferase assay, indicated that miR-544a mimic led to a marked attenuation of luciferase activity induced by LEF1-AS1-WT but not LEF1-AS1-MUT (Fig. 3b). Similarly, transfection of miR-544a mimic also resulted in a remarkable downregulation of LEF1-AS1 in A549 and H1299 cells, while miR-544a inhibitor exerted the opposing effects (Fig. 3c). Consistently, transfection of si-LEF1-AS1 significantly upregulated miR-544a expression, while the negative control showed no such effects (Fig. 3d). These data suggested the direct interaction between miR-544a and LEF1-AS1.

### Regulation of lung cancer cells by LEF1-AS1 was mediated by miR-544a

**Fig. 4:**
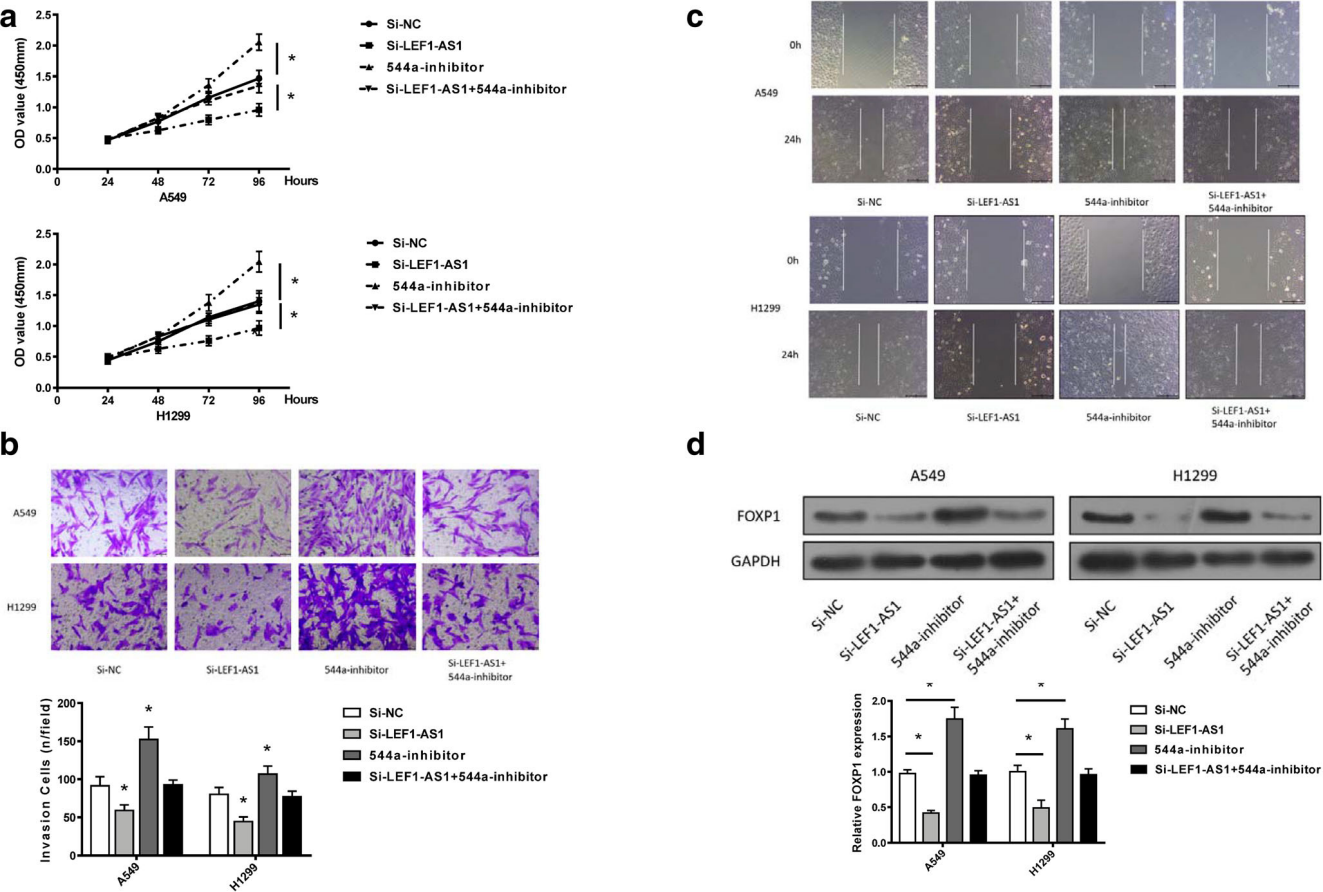
**Regulation of lung cancer cells by LEF1-AS1 was mediated by miR-544a.** CCK-8 proliferation assay (**a**), transwell assay (**b**) and scratch wound assay (**c**) n cells transfected by si-NC, si-LEF1-AS1, 544a inhibitor, or si-LEF1-AS1 + 544a inhibitor. **d**, western blot analysis of FOXP1 levels in cells transfected by si-NC, si-LEF1-AS1, 544a inhibitor, or si-LEF1-AS1 + 544a inhibitor. *p < 0.05

Next, we examined the function of miR-544a in mediating the tumor-promoting effects of LEF1-AS1 in lung cancer cells. It was found that the cells transfected with si-LEF1-AS1 presented a well-markedattenuation of cell proliferation (Fig. 4a), invasion (Fig. 4b) and migration (Fig. 4c). whereas the opposite effects were observed when miR-544a was knocked down. Notably, co-transfection with si-LEF1-AS1 and miR-544a inhibitor failed to alter the cell proliferation, invasion, and migration compared to cells without transfection. Moreover, a known effector of miR-544a, FOXP1, exhibited a negative correlation to LEF1-AS1 expression (Fig. 4d). Therefore, miR-544a plays an important role in mediating the effects of si-LEF1-AS1 in lung cancer.

### LEF1-AS1 inhibition attenuates lung cancer xenograft growth in mice

**Fig. 5:**
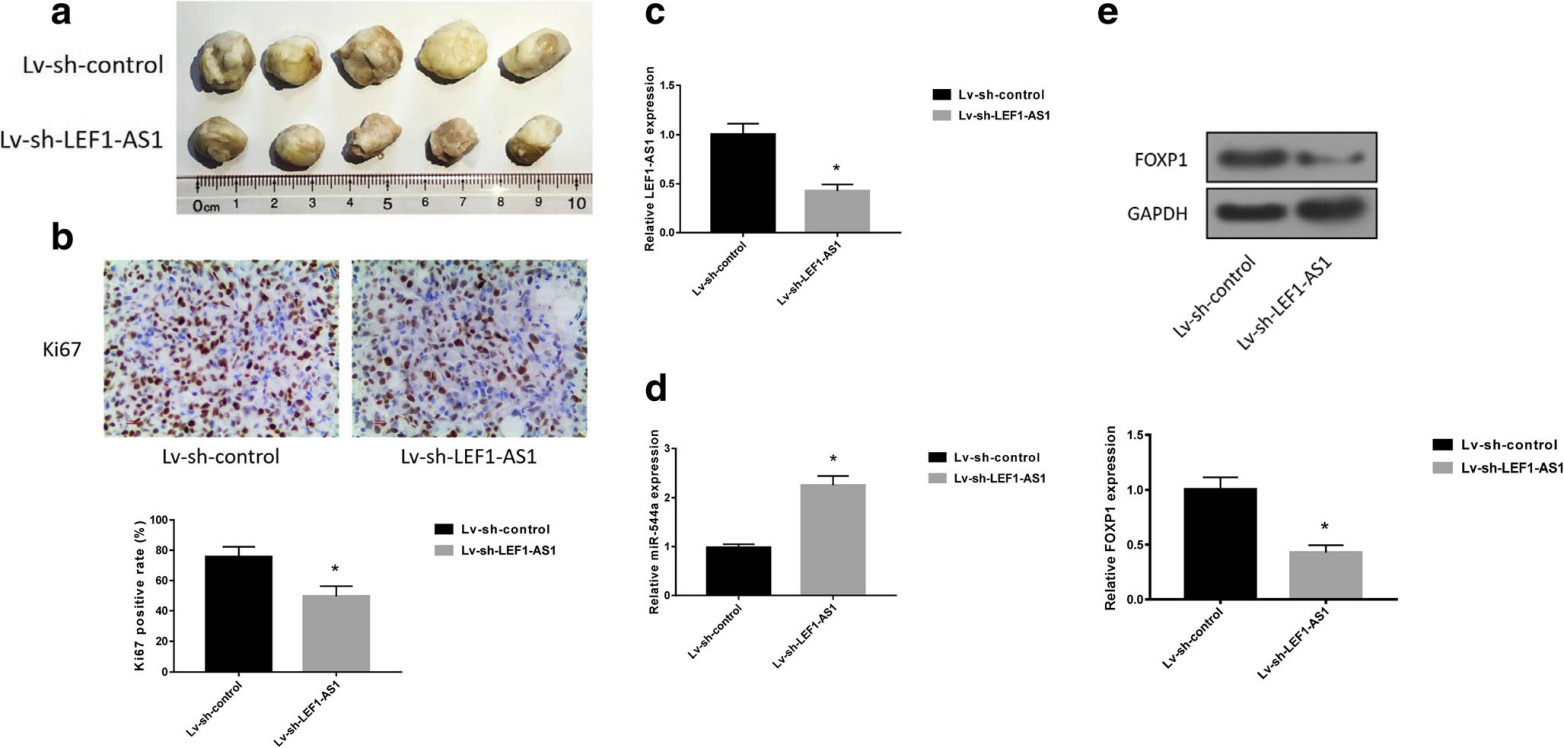
**LEF1-AS1 inhibition attenuates lung cancer xenograft growth in mice**. **a**, Visual examination of tumors initiated from A549 cells infected with lv-sh-control or lv-sh-LEF1-AS1. **b**, Representative images and quantitative analysis of Ki-67 staining of tumor sections. Expression of LEF1-AS1 (**c**) and miR-544a (**d**) were quantified by qRT-PCR. **e**, western blot analysis of FOXP1 expression in tumors. *p < 0.05

To evaluate the anti-tumor effects of LEF1-AS1 silencing in vivo, we established A549 cells stably expressing sh-control or sh-LEF1-AS1 We found that tumors with sh-LEF1-AS1 demonstrated significantly smaller sizes (Fig. 5a), as well as decreased Ki-67 expression comparing with the control (Fig. 5b).,Meanwhile, the tumors transfected with sh-LEF1-AS1 also exhibited an we obsversed obvious reduction of LEF1-AS1 expression and a prominent increase of miR-544a expression (Fig. 5c and d). Additionally, FOXP1 was downregulated in tumors with LEF1-AS1 silencing (Fig. 5e). These data validated that LEF1-AS1 silencing may be an effective strategy in inhibiting lung cancer growth.

## Discussions

In the present study, we strived to unravel the role of LEF1-AS1 in lung cancer. A previous study indicated that LEF1-AS1 acts as an oncogene in GMA but failed to identify the underlying mechanism promoting malignancy [13]. Our data in a lung cancer model reinforces the oncogenic role of LEF1-AS1 since qRT-PCR analysis revealed higher LEF1-AS1 expression in tumor tissue, compared to paired normal tissue. These differences were also confirmed in cell lines, where LEF1-AS1 was found to be significantly upregulated in a number of lung cancer cell lines, compared to a normal bronchial epithelial cell line. Several studies have recently demonstrated the link between dysregulated lncRNA expression and cancer tumorigenesis, treatment resistance, and metastasis [14–16]. The interactions between lncRNAs and macromolecules can influence multiple regulatory mechanisms of cancer either through epigenetic regulation of protein expression or direct dysregulation of lncRNAs [17]. It has been postulated that the deregulation of lncRNAs influences normal regulation of the eukaryotic genome to confer a growth advantage to cancer cells, leading to sustained and uninhibited tumor growth [18]. In support of this hypothesis, lncRNA AB073614 was shown to induce tumor progression and was associated with poor prognosis by regulating ERK1/2 and Akt signaling in ovarian cancer [19]. Additionally, lncRNA CRNDE was shown to impart pro-oncogenic abilities in gliomas by modulating mTOR signaling [20]. Several studies also indicate that lncRNAs may serve as sensitive biomarkers of specific cancer subtypes based on their cellular specificities [21, 22].

Silencing LEF1-AS1 in lung tumor cells significantly attenuated cell proliferation and invasion. After confirming the oncogenic role of LEF1-AS1, we aimed to elucidate its binding partners. Initially, BLAST analysis uncovered miR-544a as a binding partner of LEF1-AS1. MiR-544ais already a well-known inducer of epithelial-mesenchymal transition in cancer [23]. We found silencing LEF1-AS1 resulted in upregulation of miR-544a, suggesting a direct interaction between miR-544a and LEF1-AS1. This relationship was further confirmed by the negative correlation between LEF1-AS1 expression and FOXP1 expression, which is a well-known effector of miRNAs [24], Silencing LEF1-AS1 also significantly increased miR-544a expression, downregulated FOXP1 expression, lower tumor size and Ki-67 expression.

Our findings support the previous in vivo studies showing that tumors with LEF1-AS1 knockdown cells grow more slowly compared to controlsvia modulating ERK1/2 and Akt/mTOR signaling [13]. .Besides, miR-544 has been also found to interrupt adaptive responses to hypoxia via ATM-mTOR signaling [25]. LncRNA-based therapeutics are novel anti-cancer strategies that have increasingly garnered attention [26]. Understanding the underlying molecular mechanism of lncRNA therapy is of paramount importance. Through the modulation of LEF1-AS1 expression and possibly other lncRNAs, a new treatment can be formed in the fight against lung cancer and other cancers [27].

## Conclusions

In summary, the present study reveals that LEF1-AS1 is upregulated in lung cancer cell lines and tumors,which plays a positive regulatory role in lung cancer proliferation and invasion. Besides, there was a negative correlation between LEF1-AS1 and miR-544a, and FOXP1 is a downstream effector of miR-544a. Fundamentally, the LEF1-AS1/miR-544a/FOXP1 axis is an important contributor to lung cancer progression and that disrupting these signaling pathways could provide a novel mechanism for treating lung cancer.
